# Why is the market for hybrid electric vehicles (HEVs) moving slowly?

**DOI:** 10.1371/journal.pone.0193777

**Published:** 2018-03-21

**Authors:** Djamel Rahmani, Maria L. Loureiro

**Affiliations:** 1 CREDA-UPC-IRTA, Edifici ESAB, Parc Mediterrani de la Tecnologia, C/Esteve Terrades, Castelldefels, Barcelona, Spain; 2 Departamento de Fundamentos da Análise Económica, Facultade de C. Económicas e Empresariais, U. Santiago de Compostela, Santiago de Compostela, Spain; Chongqing University, CHINA

## Abstract

Hybrid electric vehicles (HEVs) could be a good short term option to help achieve global targets regarding road transport greenhouse gas emissions. Several common and country-specific public policies based on price or tax rebates are established in order to encourage the adoption of HEVs. The present research empirically assesses market preferences for HEVs in Spain, looking at the role of subsidies. An interactive internet-based survey was conducted in a representative sample (N = 1,200) of Spanish drivers. Drivers are willing to pay an extra amount of €1,645 for a HEV model compared to a conventional vehicle, premium which is well below the price markup for these cars. Therefore, current levels of economic subsidies applied in isolation to promote these types of vehicles may have a quite limited effect in extending their use. Overall, it is found that drivers have clear misconceptions about HEVs, which affect their purchasing choices and perceptions. Therefore, a policy mix of various incentives (including informational campaigns) may be required in order to stimulate the demand for HEVs.

## 1. Introduction

In December 2015, a total of 195 countries ratified a universal agreement to combat climate change at the Climate Summit in Paris (COP 21), where they expressed their willingness to move together towards a low carbon economy. The European Union (EU) has announced its plan to achieve its ambitious challenges, including the reduction of its greenhouse gas emissions by 40% by 2030 from the 1990 level; improving energy efficiency by 40%; and increasing the contribution of renewable energy in its energy consumption by 27% [1].

One of the priorities is the transport sector, due to its significant contribution to global warming, and air pollution (Directive 2009/28/EC; 2009/30/EC; Directive 2009/33/EC). In particular, road transport is a major source of greenhouse gas emissions in European cities, being responsible for one fifth of the EU’s total emissions of carbon dioxide (CO2), the main greenhouse gas. Despite a slight decrease in the last few years, these emissions are still 20.5% higher than in 1990 [2]. The consequences of air pollutants generated by the transport sector on human health are an increasing cause for concern. According to the European Commission [3], air pollution causes the premature death of more than 400,000 people in Europe every year.

However, and despite the “Dieselgate” scandal, diesel vehicles represented still 52% of all Europe’s new registrations in 2015. While in United States, Chinese, and Japanese, the new registrations in the same year were dominated by gasoline vehicles [4]. The registrations of alternative fuel vehicles (AFVs) have increased in EU member states and European Free Trade Association (EFTA) countries. In particular, Italy (14.1%), Norway (12.6%), and Poland (8.1%) registered the highest growth in the AFV new registrations in the period from 2013 to 2015 [5]. However, the growth rate of the AFV new registrations was very low in most member countries. The rise of the AFV diffusion is due to several factors, including the availability of a wide range of models in the market, but also in part to different government incentives based on direct rebates in purchase prices, tax reductions, free parking, and access to priority lanes, among others [5].

The plug-in hybrid electric vehicles (PHEV), hybrid electric vehicles (HEVs), and electric vehicles (EVs) accounted for only about 2.6% of 2015 new vehicle registrations in the EU. Thanks to the CO2-based vehicle taxation scheme, Netherlands led the PHEVs and EVs sales in 2015 with contributions of 8.8% and 0.9%, respectively. Among the EFTA countries, Norway is a clear example in terms of fiscal incentives to promote the adoption of AFVs, especially PHEVs and EVs. In fact, 22% of 2015 new vehicle sales in Norway were PHEVs and EVs [4]. Among the three models, HEVs are the most sold in EU, being the highest sales in 2015 those registered in the Netherlands (3.3%) and France (2.2%). HEVs represented 25% of 2015 Toyota vehicles sold in the EU. The HEVs are also the bet of many countries like Japan and U.S. where their 2015 market shares were around 22% and 5%, respectively [4].

Nevertheless, up to now, and despite the existence of various stimuli, the market penetration for EVs, HEVs and PHEVs is still quite low in most countries. This study explores the potential reasons behind such a low adoption rate. In particular, it explores the relevant factors that drive people’s vehicle choices, especially those that play a key role in preferring HEVs over conventional vehicles, looking not only at economic incentives but also at perceptions and knowledge about HEVs.

A HEV (non-plugin) is an AFV which uses internal combustion engines and electric batteries. It uses braking energy, which is normally wasted, to recharge the battery. HEVs offer economic and environmental advantages over conventional cars, *ceteris paribus*. The engineering literature has produced very relevant references providing accurate and technical information about the advantages in terms of current efficiency, environmental performance and future possibilities of HEVs. [6, 7, 8, 9]. In general, HEVs are cheaper than EVs and PHEVs; they do not suffer from battery problems or lack of infrastructures, and benefit from public incentives in many countries. Therefore, they should be very competitive with respect to diesel and gasoline vehicles.

It has not been clear why drivers have avoided switching to HEVs. Is it primarily related to their price? And if so, what types of incentives are needed to encourage drivers to switch to HEVs? Or is it based on other misconceptions and concerns associated with HEVs that may be more important than the price markup? Based on a discrete choice experiment (DCE) included in an extensive online questionnaire, the present paper aims to provide some insight into these questions, as well as the type of incentives that are required in order to galvanize the HEV market. Specifically, it explores preferences towards car attributes, including fuel consumption and CO2 emissions (improved in HEVs). In addition, it tests whether drivers’ perceptions towards subsidies may encourage demand for HEVs. Finally, individual heterogeneity in preferences for car attributes, including the price–a factor that is often overlooked–is considered by specifying a random parameter logit (RPL) model.

## 2. Literature review

Many contributions explored consumers’ preferences for different AFVs including EVs, HEVs, PHEVs, liquefied petroleum gas, compressed natural gas, biofuel, and hydrogen powered vehicles [10, 11, 12, 13, 14]. The results detected heterogeneous individual perceptions for different AFVs, with conventional cars remaining the most attractive option.

From the extensive existing literature on car choices, few studies [15, 16, 17, 18, 19] have specifically investigated consumers’ preferences for hybrid cars (HEVs or PHEVs). Erdem et al. [17] used a contingent valuation method to estimate the willingness to pay (WTP) for HEVs in Turkey. The results showed that people were willing to pay an average premium of US$ 858 to change to a HEV. Thatchenkery and Beresteanu [19] explored HEV demand in the USA using the United States 2006 Polk New Vehicle Registration Cross-sectional Data. They showed that people were sensitive to fuel efficiency, but were more sensitive towards horsepower and weight. Axsen et al. [15] combined the revealed preferences (recent car purchases) and stated preferences of Canadian and Californian car owners to explore how consumer preferences for HEVs have shifted (specifically focusing on the neighbor effect) as HEV market penetration increased. The results showed that the WTP for HEVs rose with the market share. Chua et al. [16] employed scales and items to compare HEV and conventional car buyers in Australia. The results from factor analysis showed that while preferences for conventional cars were more sensitive to variations in quality and performance, and less sensitive to image and social influence, HEV buyers placed a great amount of importance on their ‘green’ image and social influence, and little importance on quality and appeal. Heffner et al. [18] used informal face-to-face interviews to investigate whether the ‘green’ social image influenced United States households to adopt HEVs. The results showed that all HEV owners placed some importance on the ‘green’ image of their cars, although they did not adopt HEVs by only focusing on their image. The present research joins this line of studies by adding a number of contributions. First, it assesses the heterogeneity of preferences towards car attributes across drivers. Second, this study investigates the importance of incentives when buying efficient cars, and explores whether these incentives increase the demand for HEVs. Third, results are contextualized in the current market conditions.

## 3. Case study and policy context

The present research was conducted in Spain. The current economic crisis has resulted in the Spanish vehicle fleet being one of the oldest in Europe, currently with an average car age of 11.3 years, and with emissions that affect seriously the air quality in cities [20]. Vehicles older than 10 years account for 50% of all cars circulating in Spain [21]. Driving vehicles of this kind multiplies the environmental damage caused by road transport. In this context, various strategies have been promoted in Spain, including the *Movele* and the *Pive* public programs, both aimed at promoting the market adoption of efficient cars. The *Pive* program is designed to encourage the acceptance of HEVs, PHEVs, EVs, and extended range electric vehicles. Currently, the *Pive* program [22] offers a discount of €1,500 on the purchase of a new vehicle, after turning in a private car over 10 years of age, or any commercial vehicle over 7 years of age. The vehicle purchased must be new and an efficient model (EVs, HEVs, PHEVs, or using alternative fossil fuels). As a result of this program, the Institute for the Diversification and Energy Saving (IDAE) estimated that from 2012 to 2014 the *Pive* plan has led to the replacement of 715,000 old vehicles, saved 248 million liters of fuel per year, and reduced greenhouse gas emissions by 513,000 tons of CO2 per year [22]. The Spanish government also reformed the car registration tax (Law 34/2007 of 15th of November on air quality and protection of the atmosphere), making it inversely proportional to the amount of CO2 emissions (0% for emissions lower than 120 g/km, 4.75% of the value of the car for emissions between 120 and 160 g/km, 9.75% for emissions of between 160 and 200 g/km; and finally, 14.75% for emissions of 200 g/km or higher).

In spite of these current public policy efforts to encourage drivers to adopt HEVs, these are still not particularly popular in the Spanish market. In 2016, HEVs only accounted for 2.70% of new passenger car sales [23]. The Japanese Toyota brand led the sales of HEVs in Spain, with a market share of more than 70%. Together with its premium brand Lexus, they accounted more than 80% of the total HEVs sold in Spain in 2016. While the vast majority of HEVs sold were gasoline, diesel hybrid cars only represented 6% of the total units sold [23].

## 4. Survey design

Data from drivers were collected using an online survey directed to a representative sample of drivers over the age of eighteen. The survey was administered to 1,200 residents in Spain. The number of fully completed and useful questionnaires was 1,016. The survey asked drivers to provide information about several car related issues, including current car(s) ownership, brand preferences, awareness of energy consumption issues, and their environmental attitudes. Next, the survey provided information about HEVs, asking about their intentions and plans for future car-purchases, including a DCE to elicit preferences to buy a future car. It concluded with the socio-demographic characteristics of the driver.

Participants were asked about what size (small, medium or large) they would prefer to have their next car? And thanks to the interactive aspect of the questionnaire, this information was received immediately, and automatically, depending on their answer, they were assigned to one of the two possible versions of the DCE survey. In particular, one was designed for drivers interested in buying small or medium-sized cars and the other for those who were willing to buy large cars. A total of 875 drivers (86.12% of the completed surveys) expressed their desire to buy a small or midsize car in the future, while only 138 drivers (13.58% of the completed survey) stated their wish to adopt a large size car in the future. The survey questions were common to the participants. The only difference between the two versions was the levels of the attributes included in the DCE. In this paper, data from the survey completed by those drivers willing to buy a small and medium-size car are analyzed.

### 4.1 Experimental design and DCEs

A DCE is used as it is the more appropriate way for measuring consumer welfare, and its results are more consistent with the economic theory than a traditional conjoint analysis [24]. In addition, HEVs have a small market share, and revealed preference data sources are still scarce. The DCE method is based on the assumptions of economic rationality and utility maximization [24]. It consists of presenting drivers with several car alternatives, and asking them to choose one of them based on their preferences. Each individual is expected to choose the alternative that maximizes his/her utility. Moreover, the utility derived from an alternative is assumed to depend on the marginal utilities associated with its attributes [25]. As a HEV is a quasi-public good, both economic attributes and environmental (non-economic) attributes are included. In the survey, and prior to the DCE exercise, participants were familiarized with HEVs and the expected consumption and emissions for a mid-size car. They were also required to assume that all non-specified attributes remained constant across alternatives. A DCE was then carried out, in which the participants could select between a regular vehicle and a HEV, or just remain with the status quo option (neither car).

Focus groups, pilot surveys and previous studies were used in order to identify the most relevant attributes and suitable levels for our DCE exercise. Previous studies [13] summarized the determinant factors of a car choice process mainly into economic attributes (purchase price, fuel cost), non-economic attributes (refueling or recharging time, availability of fuel or recharging opportunities, technological performance), and environmental attributes (emissions). Besides the type of vehicle, two economic attributes have been included: price and fuel consumption, factors that are highly and primarily valued by drivers when considering the purchase of AFVs [26]. Apart from the monetary attributes, each choice set included two non-monetary or environmental attributes. The environmental attributes included were carbon dioxide (CO2) emissions, which were found to be significant in earlier studies [13], and the option of biofuel adaptation (flex-fuel), which is a recent trend in carmakers. In fact, European legislation (Directive 2003/30/EC) and national legislation (Spain’s Royal Decree 61/2006) allow carmakers to incorporate bio-fuel directly into conventional fuel without the need for specific labeling, unless the proportion exceeds 5%. Some existing studies [10, 27] have explored preferences for biofuel cars, although it has never been investigated as an additional attribute to conventional and HEVs.

The attribute levels are based on information obtained from car suppliers in the Spanish market for small and midsize cars. This information is used to determine 2 levels of vehicle type (regular or HEV) and 3 levels of prices used in the analysis: a low price level (€12,000), a medium price level (€16,000) and a high price level (€20,000). The mid-price level considered corresponds to the average price of new cars sold in Spain in 2012. From 2009 to 2013, most of the new cars sold in Spain (80% of the total) were priced below €20,000, due in part to the decrease of purchasing power of consumers caused by the economic crisis. For these reasons, and given the focus of this work (analyzing the demand for small and medium HEVs), the upper price level is set at €20,000 and the lower price level at €12,000. The fuel consumption attribute was expressed as fuel cost (€) per 100 kilometers [10, 28]. This unit is used because drivers tend to remember how much fuel their car consumes in terms of euros/kilometers. The fuel cost was computed as the product between the numbers of liters of fuel the vehicle would require to travel 100 kilometers, and the average fuel price in Spain (€1.35 per liter at the time of the study). Similarly, the CO2 emissions were expressed as grams of CO2 per kilometer [10, 28]. Again, for simplicity, and for the purposes of this research, only two emission levels are included: a more efficient level (100gr per kilometer) and an inefficient level (150gr per kilometer). Finally, the presence or absence of the potential of biofuel adaptation corresponded with the two dichotomous levels specified for the corresponding attribute.

The combination of these five attributes and their levels, using SPSS orthogonal main effects design and then the procedure of Street and Burgess [29] (vector of differences = 12111), generated an optimal orthogonal design (OOD). The OOD is constructed so as to maximize the differences in the attribute levels across alternatives, and therefore, maximize the information from each respondent, forcing the tradeoffs of all attributes in the experiment [30]. It should note that this design fits best choices where each alternative has the same number of attributes, and each attribute has the same number of levels. The final design contained 8 choice cards with a design efficiency of 98%.

**Fig 1:**
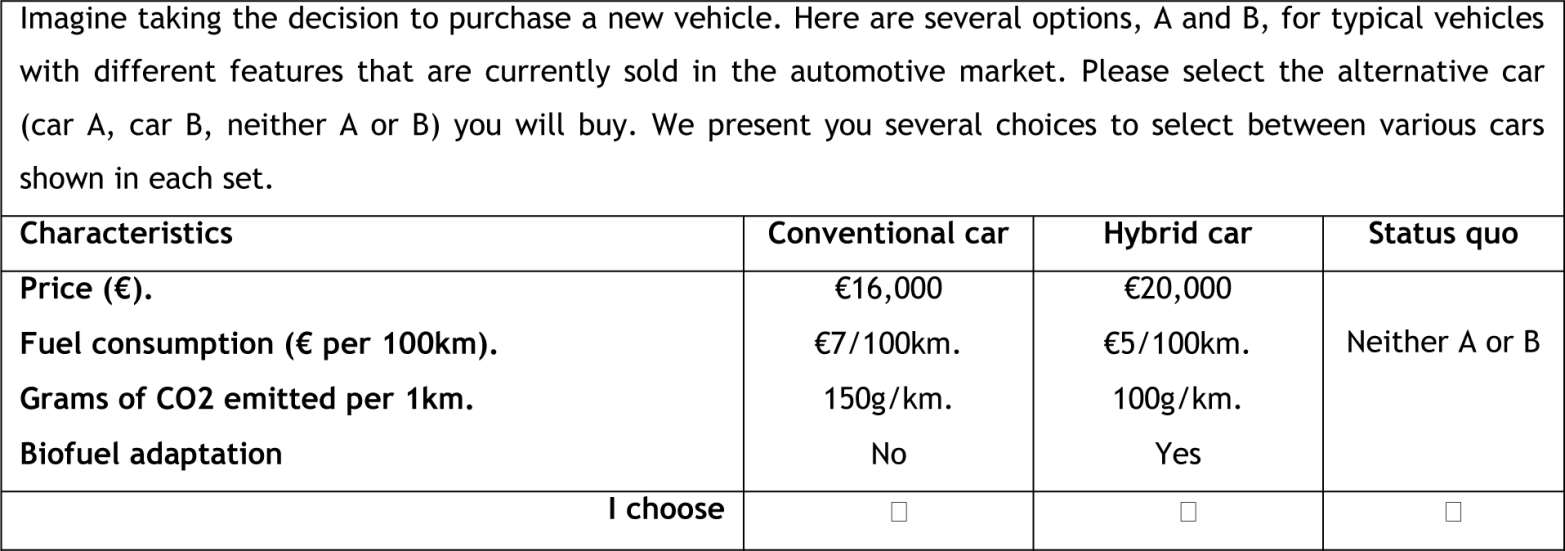
Choice experiment question and card example.

Each respondent was presented with a total of 8 choice cards, a reasonable number that does not affect data quality [31]. Fig 1 shows an example of a choice card. The no-choice alternative (neither car) was provided in order to make the choice decisions very similar to market decisions (or more realistic).

### 4.2 Choice modeling specification

Assuming utility maximizing behavior, the empirical applications based on discrete choice models make possible to estimate the probability that an individual chooses a given car alternative, among a set of available alternatives. The utility that an individual i derived from choosing a car alternative j among a set of J alternatives (conventional car, HEV or no-choice option) in each choice situation t may be expressed [32, 33] as a sum of an observable utility component (${\text{X}}_{\text{ijt}}^{\text{'}}\beta$) and unobservable component or error term (ε_ijt_):

$${\text{U}}_{\text{ijt}}{\text{=X}}_{\text{ijt}}^{\text{'}}\beta +\varepsilon_{\text{ijt}}$$ (1)

where,

${\text{X}}_{\text{ijt}}^{\text{'}}$: is a vector of specific car attributes and specific individual characteristics.

β: is a vector of parameters associated with the explanatory variables.

The multinomial logit model (MNL) [34] is derived assuming that the error terms (ε_ijt_) are independently and identically extreme value type I distributed (IID). The MNL probability of choosing an alternative a among a set of J alternatives is given by [32, 33]:

$$L_{\mathrm{iat}}=\frac{\exp(X_{\mathrm{iat}}^{'}\beta )}{\overset{J}{\underset{j=1}{\sum }}\exp(X_{\mathrm{ijt}}^{'}\beta )}$$ (2)

The MNL is based on the assumption of the independence of irrelevant alternatives (IIA). The MNL imposes homogeneity in tastes, inflexible substitution patterns in preferences between different alternatives and independence in unobserved factors over time [35]. An alternative model which is much more flexible and which overcomes the limitations of MNL is the RPL. In addition to the fact of not complying with the IIA property, the RPL allows for: a) random heterogeneous preferences across individuals, b) unrestricted substitution patterns, and c) correlation in unobserved factors over time [35]. The RPL model probability (unconditional probability) is the integral of the conditional probability over all the random parameters [32, 33]:

$$P_{\mathrm{iat}}=\int \frac{\exp(X_{\mathrm{iat}}^{'}\beta )}{\overset{J}{\underset{j=1}{\sum }}\exp(X_{\mathrm{ijt}}^{'}\beta )}f(\beta |\theta )d\beta$$ (3)

where,

f(β|θ): is the density function of the parameters β. This density function may be assumed to follow any closed-form distribution (normal, log-normal, triangular, uniform) [36, 35]; θ: are the parameters (mean and standard deviation) of the distribution.

In this application, RPL models are estimated assuming log-normal distribution for the coefficients associated with price (PRICE), fuel consumption (FCONSUMPTION) and CO2 emissions (CO2) in order to force them to be negative (on one side of zero) for all individuals. In the same line as previous studies [10, 37, 12], positive preferences for these attributes are not allowed, as it is not expected that people would prefer higher prices, higher fuel consumptions or higher CO2 emissions. Several distributions (normal, log-normal, uniform, triangular, etc.) are also tested for the coefficient associated with biofuel adaptation (BADAPTATION) but its standard deviation was not statistically significant. Thus, it is considered as a nonrandom parameter. This valuation exercise also aims to predict respondents choices between the two car-alternatives (conventional, and HEVs) and the no-car option (neither A or B), including in the DCE models a no-choice-specific constant (ASC), denoting the election of the status quo option. It is assumed that the no-choice-specific constant follow a normal distribution because drivers may like or dislike staying or not with their current cars. In addition, it explores how preferences for the no-car option, compared to the car options, shift with the following socioeconomic variables: gender (MALE), age (AGE), and monthly income under €1,800 (LHINC). It also analyzes the heterogeneity in preferences for the no-car option among drivers who reported that incentives such as direct subsidies (SUBSIDY) would be important factors when buying an efficient car. This incentive variable was created from the participants’ ratings, when they were asked to state how important (on a 5-point Likert scale: from 1 “not important” to 5 “very important) this factor would be in their decision to select efficient cars.

Furthermore, and after the vector of parameters is obtained, the WTP welfare measures are calculated in order to determine the monetary equivalent of the marginal utilities placed by drivers in each car attribute improvement. This step may provide important information to policy makers regarding the economic efforts that people are willing to make to acquire HEVs and some improvements in car attributes.

WTP for a HEV compared to conventional vehicle is generally computed as the difference (MU_HEV_ ‒ MU_C_) between the marginal utility obtained for HEV (MU_HEV_) and conventional vehicles (MU_C_). Such values are obtained substituting in (1) the estimated parameters of our empirical model, and then this difference in utilities is divided by the estimated price coefficient (β_PRICE_) [33], as shown by the following formula:

$${\mathrm{WTP}}_{\mathrm{HEV}}=-\frac{\left({\mathrm{MU}}_{\mathrm{HEV}}-{\mathrm{MU}}_{C}\right)}{\beta_{\mathrm{PRICE}}}$$ (4)

In a RPL model, when the numerator and the denominator included in Eq 4 are random, the expression of the WTP ratio shown in Eq 4 becomes a randomly distributed term. In this case, Daly et al. [38] advised to ensure finite moments for the WTPs. In the present application, WTP measures are constructed based on unconditional parameter estimates [39], because they allow for prediction outside of the sample, unlike conditional parameter estimates, which only predict within the sample [40]. Deriving WTP based on unconditional parameter estimates requires the population to be simulated [39]. Both, numerator and denominator of (4) have been simulated employing random draws coming from the log-normal distributions defined by the estimated parameters. Draws were generated from both, the numerator and denominator, computing their respective ratio in each draw, as in Hensher et al [39].

## 5. Data and results

Table 1 summarizes the drivers’ perceptions towards HEVs (prior to the information received in the survey, given that participants could select several statements that they considered correct when thinking about HEVs). When compared to conventional cars, 62% of the drivers perceived HEVs as being more expensive, although 28% stated that HEVs have low running costs. In addition, 14% believed that HEVs are slower, and 18% considered that HEVs have less power. These negative perceptions can be an obstacle to introduce HEVs in a country where drivers are in general “speed lowers”. Furthermore, it is worrisome that 16% reported that they did not know what HEVs are like. Finally, 17% reported that HEVs have limited autonomy, showing a clear misunderstanding of the difference between HEVs and EVs.

**Table 1:** Perceptions for hybrid electric vehicles (HEVs).

Participants’ hybrid car Perceptions (1 = yes, 0 = no)	Mean	Std. Dev.
**Compared to a conventional car:**		
**A hybrid car is more expensive**	.620	.485
**A hybrid car has lower running costs**	.281	.449
**A hybrid car is slower**	.142	.349
**A hybrid car has less autonomy**	.168	.374
**A hybrid car is less powerful**	.176	.381
**A hybrid car is less safe**	.013	.116
**I do not know what a hybrid car is**	.158	.365

Table 2 describes some drivers’ socio-demographic characteristics and the variables included in the empirical models, containing basic information about the rated importance of incentives including direct subsidies, registration tax exemption, free parking, access to priority lanes, and social image. In summary, public policies based on direct economic incentives, such as subsidies or allowing free parking are perceived as the most important incentives for drivers to buy an efficient car.

**Table 2:** Descriptive statistics of the variables included in the RPL model.

Variable	Description	Mean	Std. Dev.
**PRICE**	price of car-option divided by 10,000.	1.066	.805
**FCONSUMPTION**	euros spent in fuel consumption per 100km.	4	2.943
**CO2**	grams of CO2 emitted per 1km.	83.333	62.362
**BADAPTATION**	1 if car-option is adaptable (flex-fuel) to run with biofuels and 0 otherwise.	.333	.471
**ASC**	no-car-option constant.	.333	.471
**MALE**	1 for male and 0 otherwise.	.513	.499
**AGE**	age of participants (years).	45.972	13.546
**LHINC**	1 for monthly income under €1,800 and 0 otherwise.	.505	.499
**SUBSDY**	importance (score) attributed to the incentive “direct subsidies”.	4.199	.961

In terms of socio-demographics, the average age of participants in this sample is 46 years, and 51% of the participants were male. One fifth of the participants were unemployed, and about 50% of all households received a monthly income of less than €1,800. The participants reported that on average they drive a car 4 days a week. The sample was representative of the profile of a Spanish driver at least with respect to some important characteristics, such as age and driving frequency. The Spanish Observatory of Drivers [41] defined, through a representative study, a typical Spanish driver as being a 44-year-old male, who uses a car an average of 5 days a week for work.

Table 3 summarizes the results of the estimated models. First, a MNL model is estimated and the assumption of independent irrelevant alternatives (IIA) is tested using the Chi-squared Hausman and McFadden test. The results of this test reject the IIA assumption [being the omitted alternative the regular car: Chi-squared (5) = 156.808; with the omitted alternative being the HEV: Chi-squared (5) = 160.883; the 99%; critical value: Chi-squared (5) = 15,086]. Then, to improve the performance, RPL models have been estimated, allowing for correlation over time (but with uncorrelated parameters) and using NLOGIT.5 software with 2000 replication draws in the estimation processes. In particular, a baseline RPL and a RPL model with heterogeneity in the mean of the random parameter associated with the no-car specific constant are specified.

**Table 3:** Results of estimated MNL and RPL models.

	MNL			Baseline RPL			RPL		
Parameters in utility functions									
	Coeff.	Std. Error	Prob. |z|>Z	Coeff.	Std. Error	Prob. |z|>Z	Coeff.	Std. Error	Prob. |z|>Z
**PRICE**	-2.034	.055	.000	-2.450	.059	.000	-2.371	.051	.000
**FCONSUMPTION**	-.289	.017	.000	-.336	.024	.000	-.348	.023	.000
**CO2**	-.009	.001	.000	-.013	.001	.000	-.014	.001	.000
**BADAPTATION**	.157	.033	.000	.154	.041	.000	.148	.041	.000
**ASC**	-6.017	.178	.000	-8.175	.220	.000	-9.487	.372	.000
**Standard deviations of random parameters**									
**LSPRICE**	.			.832	.063	.000	.621	.035	.000
**LSFCONSUMPTION**	.			.150	.023	.000	.130	.019	.000
**LSCO2**	.			.012	.002	.000	.012	.0004	.000
**NSASC**	.			2.193	.109	.000	1.209	.080	.000
Heterogeneity in mean, Parameter * Variable									
ASC * MALE	.			.			-.620	.114	.000
ASC * AGE	.			.			.019	.004	.000
ASC * LHINC	.			.			.199	.111	.074
ASC * SUBSDY	.			.			.161	.049	.001
**Measures of goodness of fit**									
**N**	7,000			7,000			7,000		
**GROUPS**	875			875			875		
**NB. OBS./GROUP**	8			8			8		
**L.L. FUNCTION**	-6,655.621			-5,528.428			-5,284.316		
**K (factors number)**	5			9			13		
**CHI SQU. [K]** **SIGNIFICANCE**	. .			4,323.715 .000			3,933.048 .000		
**R-SQUARED**	.126			.281			.271		
**ADJ. R-SQUARED**	.125			.281			.270		
**AIC**	13,321.2			11,074.9			10,594.6		

In Table 3, Column 1 shows the results of the MNL; the RPL results are presented respectively in Column 2 (baseline RPL), and Column 3 (extended RPL, with interaction terms with the constant). According to the values of the log-likelihood, adjusted pseudo-R^2^, Akaike information criterion (AIC), the RPL improves the MNL model fit (which results are not directly discussed). The moments of the coefficients associated with PRICE, FCONSUMPTION, and CO2 which are calculated converting the log terms are presented in this table.

The results of the baseline RPL model show that the mean of both nonrandom and random parameters are significant and have the expected signs. The sign of the variables PRICE, FCONSUMPTION, and CO2 have been inversed and entered as negative in the RPL model specifications. The log-normally distributed coefficients are expressed as: β_k_ = exp(b_k_+s_k_μ_k_) where, μ_k_ is IID standard normal deviate, b_k_ and s_k_ are the estimated mean and standard deviation for log-normally distributed coefficient. The table above presents the moments of the coefficients associated with PRICE, FCONSUMPTION, and CO2 which are calculated in the following way [42, 43]: $\mathrm{Mean}=\exp(b_{k}+(s_{k}^{2}/2))$; $\text{Standard deviation}=\mathrm{mean}*\sqrt{\left(\exp(s_{k}^{2})-1\right)}$. The mean of PRICE, FCONSUMPTION, and CO2 were multiplied by -1 in order to reestablish the sign changed a priori to the model estimation.

The results are similar to those provided by the MNL model. In line with previous findings [26], the two monetary attributes that were included–price (PRICE) and fuel consumption (FCONSUMPTION)–have negative effects on utility, indicating that drivers tend to prefer cars with a lower price and lower fuel consumption. This implies that one of the motives that may attract drivers to adopt HEVs is fuel economy. Similarly, and in agreement with findings from previous studies [13], the coefficient associated with CO2 emissions (CO2) is negative, implying that on average, drivers prefer cars with lower levels of CO2 emissions. Furthermore, the fact that the car could be adapted to biofuels (BADAPTATION) carries a positive effect on utility, implying that drivers prefer flexible-fuel cars to non-adapted cars. Regarding the standard deviations, it is found that the three random parameters (PRICE, FCONSUMPTION, and CO2) have statistically significant standard deviations, implying that there are heterogeneous preferences for these attributes across drivers. Moreover, the no-car option constant is negative and statistically significant at any critical level. This reveals that drivers prefer to move from status quo to any of the two car type options. The standard deviation of the constant (ASC) is also statistically significant suggesting that drivers preferences for their actual cars are heterogeneous. In order to control for the potential wide heterogeneity of preferences towards actual cars, it is considering the next extended RPL model.

The next RPL model explores whether the shift in the mean of the no-car-specific constant due to drivers’ socio-demographic characteristics (MALE, AGE and LHINC) and drivers’ preferences towards the incentives (SUBSIDY) when buying efficient cars, improves the fit of the baseline RPL. The results show that MALE are less likely to choose the status quo no-car option over an efficient car in terms of consumption (HEV or a conventional car) in the DCE, whereas older drivers (AGE) prefer staying at the status quo option. Drivers with monthly income under €1,800 show significant preferences for their actual cars. Interaction term between the no-car-option constant and the preferences towards direct incentives via subsidies is positive and statistically significant, indicating that the more important subsidies (SUBSDY) are for drivers, the more likely they are to stay with the no-car status quo option compared to enter the market of HEVs or conventional vehicles. These may be caused by the fact that drivers who are more sensitive to subsidies are also more sensitive to price in general, and as a consequence, they are not willing to enter the HEV market, which is more expensive. This finding suggests that drivers do not know that HEVs are supported with economic subsidies (*Pive* Plan) or they think that these subsidies are not enough to afford a HEV.

Table 4 shows the sample mean WTP for the HEV compared to conventional car estimated from the basic RPL empirical model. To estimate this WTP, we used the characteristics of a concrete example of HEV and its conventional model of the Toyota brand. Table 4 shows these characteristics for both models. Results show that drivers are willing to pay a statistically significant premium of €1,645.032 to move from the conventional model to the HEV. Therefore, participants are willing to make an economic effort to buy a HEV compared to conventional car, although this is well below the price markup for these cars. Therefore, current levels of economic subsidies (*Pive* Plan) applied in isolation to promote these types of vehicles may have a limited effect in extending their use.

**Table 4:** Mean WTP for HEV compared to conventional vehicle.

Attribute	HEV	Conventional model	WTP	
**Price (PRICE)**	€20,200	€18,550	Mean:	€1,645.032
			Std.Dev.:	€5.319
**Fuel/100Km.(FCONSUMPTION)**	3.6l	5l	Median:	€1,646.7
			1st.Qrtl:	€1,609.1
			3rd.Qrtl:	€1,636.2
**CO2 emissions per 1km (CO2)**	75gr	112gr		

Once again, the results reiterate the need of informational mechanisms as to ensure the understanding of HEVs attributes and the incentives supporting the use of HEVs. This is in fact nowadays an obstacle to increasing the market share of hybrid technology.

## 6. Conclusions and implications

The present research explores the importance of monetary and non-monetary incentives, generally adopted by governments to boost sales of fuel-efficient cars. It tests whether drivers who consider these incentives to be important are especially attracted to HEVs. Participants in a national survey were asked to rate the importance that some public policies would have in their decision to switch to efficient cars (HEVs or new medium cars). They rated policies based on reductions in direct subsidies or allowing free parking to be the most important incentives, followed by access to priority lanes and registration tax exemption, and finally by the social image derived from driving an efficient car.

Nearly half of the sample perceives HEVs to be cleaner than gasoline, diesel, biofuels, and liquefied petroleum gas (LPG) cars. Three out of ten drivers believe that HEVs have lower running costs than conventional cars. However, many drivers (62%) perceive them to be more expensive, slower (14%), and less powerful than conventional cars (18%). In addition, some drivers do not exactly know what HEVs are (15%), or clearly misunderstand the difference between HEVs and EVs (16%).

The estimated stated preference models show that drivers prefer cheaper cars with low fuel consumption, implying that fuel economy may be an attractive reason to buy HEVs. Similarly, low CO2 emissions increase the utility derived from a car, and are another reason to encourage drivers to buy HEVs. The subjects also expressed strong preferences for flexible-fuel cars, concluding that offering HEVs adapted to run with biofuels could increase the demand for HEVs.

The results derived from the RPL model show that drivers are willing to pay an extra premium of €1,645 to change from conventional automobiles to HEVs; however this amount is well below the price markup for these cars in the current market. Therefore, current levels of economic subsidies applied in isolation to promote these types of vehicles may have a limited effect in extending their use.

In this context, the measures implemented in countries such as Norway and France to encourage adoption of EVs have already demonstrated their effectiveness and may be considered by third countries. In particular, Norway reached the target of 50,000 EVs in 2015 through a mix of public monetary and non-monetary incentives in favor of EVs, including VAT and registration tax exemption, access to priority lanes, free toll roads, free parking, free travel on ferries, free municipal recharging, reductions in the annual road tax, and exemption from company car tax [44]. The *bonus-malus* French system was able to reduce the emissions of newly registered passenger vehicles by 19g/km in just three years after its introduction [45]. This system offers a bonus of €6,000 (up to 27% of the acquisition cost) for the purchase of a new private car or van that emits up to 20 grams of CO2/km and a bonus of €2,500 for handing over an old diesel vehicle put into circulation before January 1, 2006. These two financial aids are cumulative, therefore the total aid may reach €8,500. Moreover, it imposes a tax up to €10,500 for the purchase of vehicles emitting more than 185 grams of CO2/km [46]. Furthermore, other restrictive measures (access to the city center, surroundings, or to parking) used by some large European cities to deal with the emergency pollution situations could encourage drivers to opt for AFVs if they were applied more frequently (a few days a week).

To conclude, and as earlier stated, the articulation of economic incentives may be crucial. Further, designing information campaigns that provide accurate information on HEVs may have as well a significant impact on sales. Another potential solution is to promote the use of HEVs for taxis and public transport, and to encourage the public authorities to replace their conventional cars with HEVs. This may help to further promote the image of HEVs, and to reduce the current distrust towards this alternative fuel technology. Various governments (Japan, EU, Canada, China, South Korea, Mexico, Brazil, and India) have fixed greenhouse gas emission limits for passenger vehicles to meet short-term mitigation goals. In particular, EU fleet target for 2021 is 95g/km [47], which is equivalent to the amount of CO2 emitted by various HEVs currently available on the market. In this context, encouraging the adoption of HEVs will not only reduce the compliance deadline but also the cost required. This set of findings may be relevant in order to adopt appropriate and effective strategies in the future aimed at reducing road transport, greenhouse gas emissions, and their contribution to climate change. Future research should look deeper at the role of economic incentives under different scenarios, characterized in many occasions by strong cultural differences, risk aversion, and myopic time preferences.
